# Leveraging core enzyme structures for microbiota targeted functional regulation: Urease as an example

**DOI:** 10.1002/imt2.70032

**Published:** 2025-04-16

**Authors:** Shengguo Zhao, Huiyue Zhong, Yue He, Xiaojiao Li, Li Zhu, Zhanbo Xiong, Xiaoyin Zhang, Nan Zheng, Diego P. Morgavi, Jiaqi Wang

**Affiliations:** ^1^ Key State Laboratory of Animal Nutrition and Feeding, Institute of Animal Sciences Chinese Academy of Agricultural Sciences Beijing China; ^2^ Electron Microscopy Centre Lanzhou University Lanzhou China; ^3^ Université Clermont Auvergne, INRAE, VetAgro Sup, UMR Herbivores, Saint‐Genès‐Champanelle Clermont‐Ferrand France

**Keywords:** core, epiberberine, function, microbiome, protein structure, rumen, urease

## Abstract

Microbial communities play critical roles in various ecosystems. Despite extensive research on the taxonomic and functional diversity of microbial communities, effective approaches to regulate targeted microbial functions remain limited. Here, we present an innovative methodology that integrates core enzyme identification, protein structural characterization, regulator virtual screening, and functional validation to achieve precise microbiome functional regulation. As a proof of concept, we focused on the regulation of urea decomposition by the rumen microbiota in ruminants. Through metagenomic analysis, we identified the core urease gene and its corresponding microbial genome (MAG257) affiliated with the unclassified Succinivibrionaceae, and reconstructed its complete gene cluster. Structural analysis of the urease catalytic subunit (UreC) via cryo‐electron microscopy (cryo‐EM) revealed detailed features of its active site, guiding molecular docking studies that identified epiberberine, a natural compound with potent urease inhibitory activity. Validation in a rumen simulation system demonstrated that epiberberine significantly reduced urea decomposition and enhanced nitrogen utilization. This study establishes a robust framework that combines structural biology and computational screening to achieve targeted microbiome functional regulation, offering a promising tool for microbiome engineering and broader applications in animal productivity, human health, environmental improvement, and biotechnology.

## Highlights

- Despite extensive research on the taxonomic and functional diversity of microbial communities, effective approaches to regulate targeted microbial functions remain limited.
- We established a robust framework that combines structural biology and computational screening to achieve targeted microbiome functional regulation.
- It offers a promising tool for microbiome engineering and broader applications in animal productivity, human health, environmental improvement, and biotechnology.

## Graphical abstract

We present an innovative methodology that integrates core enzyme identification, protein structural characterization, regulator virtual screening, and functional validation to achieve precise microbiome functional regulation. As a proof of concept, we focused on the regulation of urea decomposition by the rumen microbiota in ruminants. This methodology can be used for microbiome engineering and broader applications in animal productivity, human health, environmental improvement, and biotechnology.

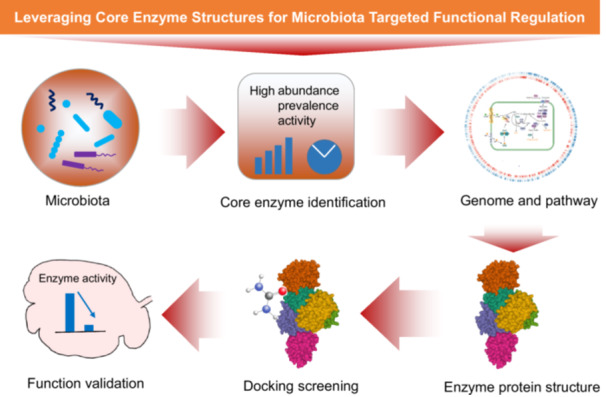

## INTRODUCTION

Microbial communities are fundamental drivers of biochemical processes across diverse ecosystems, ranging from human and animal hosts to soil, oceans, and other environments. These communities regulate a multitude of essential functions, including nutrient cycling, energy production, and metabolic homeostasis [1, 2]. While significant progress has been made in understanding the taxonomic and functional diversity of microbial communities, translating this knowledge into actionable strategies for precise regulation of microbial functions remains a major scientific challenge [3, 4]. Traditional microbiome modulation approaches, such as broad‐spectrum antibiotics, probiotics, or dietary interventions, often result in unintended perturbations, affecting nontarget microbial functions [5]. They may also produce inconsistent results due to off‐target effects, and negatively impact the normal microbial ecosystem. To overcome these limitations, there is an increasing need for targeted strategies that manipulate specific microbial functions without disrupting the overall ecosystem stability.

One promising avenue lies in the regulation of microbial enzyme activity, as enzymes represent the functional executors of microbial metabolism [6]. Enzymes such as urease, cellulase, and protease catalyze pivotal reactions that underpin microbiome‐driven processes. Targeting specific enzymes through inhibitors, activators, or structural modifications offers a direct and precise method to influence microbiome functionality. Due to the diversity of microbiomes, the gene composition and protein structure of an enzyme vary among different microorganisms [7, 8]. It is essential to identify core enzymes within the microbiome as regulatory targets to maximize the regulation of microbiome function. Core enzymes should exhibit characteristics such as high abundance, prevalence, and activity within the microbiome. Building upon this foundation, employing techniques like crystallography or cryo‐electron microscopy to elucidate the protein structures of core enzymes, rather than relying solely on software‐based structural predictions, provides a crucial basis for molecular docking and screening effective binding compounds, thereby enabling effective microbiome function regulation. However, leveraging core enzyme structure‐function relationships to design such regulatory strategies has been underexplored in the context of microbiome research.

It is well known that meat and milk from ruminant animals play an important role in human nutrition and food security around the world [9]. Urea is a common source of non‐protein nitrogen (NPN) to replace plant protein in the diet of ruminants [10]. Urea promotes ruminal fermentation and is a source of nitrogen for the rumen microbiota to synthesize microbial protein, which is then used by the host [11]. However, the rate of urea hydrolysis is much faster than that of ammonia utilization by ruminal microorganisms resulting in low utilization efficiency of urea, potential ammonia toxicity, and excess nitrogen losses to the environment [12]. Ruminant production is also one of the major sources of global nitrogen emissions [13, 14], and contributes to almost 30% of the global total ammonia emissions [15]. It is, therefore, beneficial for ruminant production and the environment to slow down the rate of urea hydrolysis so as to improve nitrogen utilization in the rumen. In the rumen, urea hydrolysis is catalyzed by urease produced by ureolytic bacteria. Urease genes are diverse in the rumen microbial community, and approximately 67% of them could not be classified at the genus level, indicating their low identity to the known urease genes [16]. The number of polypeptide chains that form the urease functional unit also varies between bacteria [17]. It is not easy to obtain efficient inhibitors regulating the whole rumen microbiota if only targeting certain known urease. Therefore, it is essential to identify the core urease and screen inhibitors based on their structure to regulate the urease of whole rumen microbiota for increasing urea utilization efficiency.

We hypothesized the existence of novel plant‐derived inhibitors of rumen urease that would be universally active, independent of diet, farm, and individual animal. We first identified the core microbial urease from the ruminal microbial community of dairy cows by sequencing, then revealed the protein structure of urease, and used the information to screen plant‐derived inhibitors by molecular docking. Finally, the found inhibitor was verified by the rumen simulation system.

## RESULTS

### Diversity of the microbial *ureC* gene in the rumen

**Figure 1:**
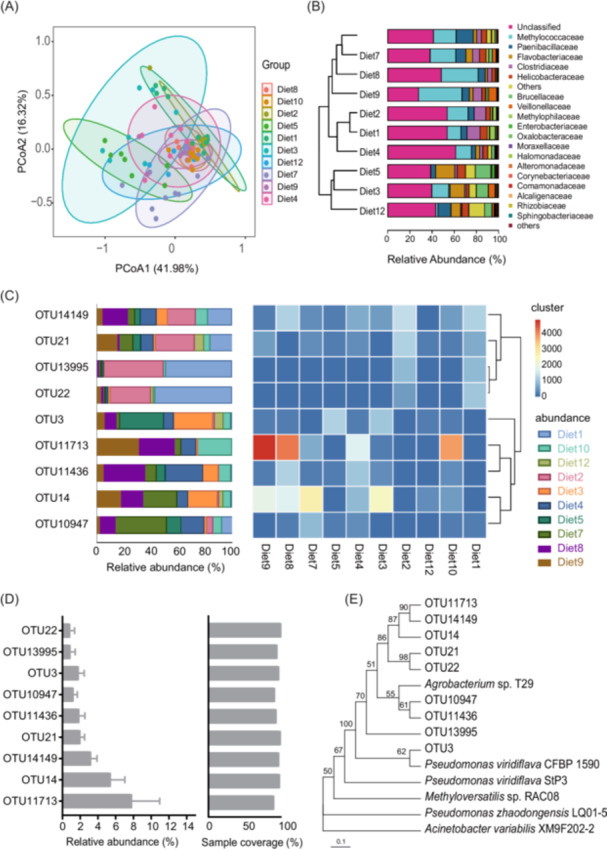
Identification of core *ureC* operational taxonomic units (OTUs) in the rumen microbiota. (A) Principal coordinate analysis (PCoA) of *ureC* genes across different diets, with different colors representing distinct diets. (B) The relative abundance of species in various diets and clustering of diets based on taxonomic annotation. (C) Clustering and relative abundance of nine core *ureC* OTUs across ten diets. (D) Relative abundance of core *ureC* OTUs across different diets, with sample coverage ratios for each core *ureC* OTUs. (E) Phylogenetic analysis of the nine core *ureC* OTUs.

A total of 88 rumen fluid samples were collected from dairy cows fed 10 different diets, producing 0.75 Gbp of clean data of *ureC* gene. The average number of reads per sample was 37,364, and the average *ureC* gene length was 271 bp. At a 97% identity threshold, 7118 *ureC* operational taxonomic units (OTUs) were identified. Principal coordinates analysis was used to compare the *ureC* gene diversity profiles across different diets. As shown in Figure 1A, distinct clustering patterns were observed based on diet. Samples from diet2, diet4, diet8, diet9, and diet10 formed tightly clustered groups, while those from diet1, diet3, diet5, diet7, and diet12 were more dispersed.

The five most dominant *ureC*‐containing families were Methylococcaceae (17.91%), Paenibacillaceae (6.92%), Flavobacteraceae (5.75%), Clostridiaceae (5.19%), and Helicobacteraceae (4.86%) (Figure 1B). On average, 44.6% of *ureC* OTUs belonged to unclassified taxa at the family level, indicating the presence of numerous unidentified and novel microbial urease genes in the rumen. Based on family‐level species composition, the diets could be grouped into three distinct clusters (Figure 1B): Group 1 (diet1, diet2, and diet4) had over 50% of *ureC* OTUs belonging to unclassified families; Group 2 (diet10, diet7, diet8, and diet9) was dominated by Methylococcaceae and Paenibacillaceae, along with unclassified families, which together comprised more than 70% of the total relative abundance; Group 3 (diet3, diet5, and diet12) exhibited a higher diversity of dominant families, with five major taxonomic groups contributing over 70% of the total relative abundance, excluding unclassified families.

### Identification of ruminal microbial core *ureC* gene

The number of *ureC* OTUs that appeared in different proportions of samples was calculated. As the proportion of samples increased, the number of shared OTUs decreased markedly (Figure S1). Only two OTUs were present in all samples, nine OTUs were detected in 95% of samples, and 30 OTUs were found in 90% of samples (Figure S1). Despite only 110 OTUs (1.55%) being present in 70% of the samples, these OTUs accounted for 52.6% of the total relative abundance. Interestingly, some of the top 30 most abundant OTUs were present in less than 50% of the samples.

Core *ureC* OTUs were defined based on a relative abundance greater than 1% and prevalence over 85% of samples. Nine core *ureC* OTUs were identified, in which OTU14149 and OTU14 were the top two abundant OTUs, with relative abundances of 6.15% and 7.89%, respectively (Figure 1C). The sample coverages for these nine OTUs ranged from 89.77% to 100%. Phylogenetic analysis indicated that these nine core OTUs did not cluster with known urease genes. The three most abundant core OTUs including OTU11713, OTU14149, and OTU14 clustered together, forming a distinct clade (Figure 1D). Clustering analysis of the 10 diets and the nine core *ureC* OTUs revealed that all the OTUs instead of OTU11713 showed small variation among diets (Figure 1E). In terms of distribution, samples from diet2 and diet11 had higher relative abundances of OTU22 and OTU13995, while their distribution across other core OTUs was more balanced.

### Identification and characterization of genomes harboring core *ureC* genes

**Figure 2:**
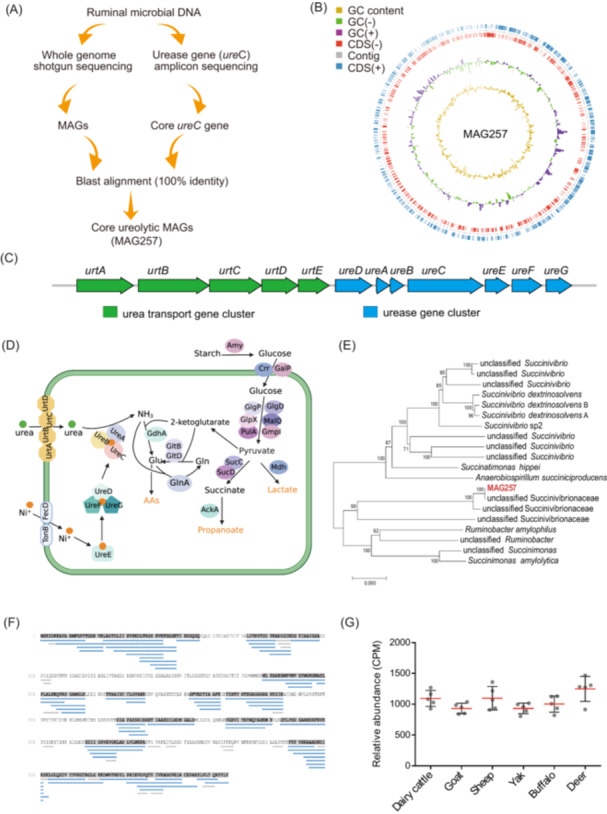
Genomic characterization of MAG257. (A) Diagram illustrating the identification process of core MAGs with urease genes. (B) Circular genome representation of MAG257. From the outer to the inner circles: CDS(+) (blue), contig (gray), CDS(−) (red), GC content positive (purple), GC content negative (green), and overall GC content (brown). (C) Gene clusters responsible for urea catalysis (blue) and urea transport (green). (D) Schematic diagram of proteins involved in urea and carbohydrate metabolism in MAG257. (E) Phylogenetic tree showing MAG257's relationship to other species within the Succinivibrionaceae family. (F) Validation of MAG257 urease with high ureolytic activity using activity‐based metaproteomics. The blue line represents peptides with high confidence, the gray line represents peptides with low confidence, and the gray background represents identified amino acids. (G) Relative abundance of MAG257 across different ruminant species, including dairy cattle, goats, sheep, yaks, buffaloes, and deer. The red line represents the mean, and the black line represents the standard deviation. CDS, coding sequence; GC, guanine‐cytosine; UrtA, urea transporter A; UrtB, urea transporter B; UrtC, urea transporter C; UrtD, urea transporter D; TonB, TonB protein; FecD, ferric citrate transport protein D; UreA, urease A; UreB, urease B; UreC, urease C; UreD, urease D; UreE, urease E; UreF, urease F; UreG, urease G; GdhA, glutamate dehydrogenase A; GltB, glutamate synthase B; GltD, glutamate synthase D; GlnA, glutamine synthetase A; Glu, glutamate; Gln, glutamine; Amy, amylase; Crr, carbohydrate transport system component; GalP, galactose permease; GlgP, glycogen phosphorylase; GlpX, fructose‐1,6‐bisphosphatase; PulA, pullulanase; GlgD, glycogen debranching enzyme; MalW, maltose transport system protein; Gmpl, guanosine monophosphate synthetase; SucC, succinyl‐CoA synthetase C; SucD, succinyl‐CoA synthetase D; AckA, acetate kinase; Mdh, malate dehydrogenase.

Alignment between metagenome‐assembled genomes (MAGs) and core *ureC* gene OTUs revealed that only MAG257 matched core OTU14 with 100% identity, identifying it as the core ureolytic bacterial genome (Figure 2A). Genome analysis of MAG257 showed a completeness of 91.48% and a contamination of 1.72%. MAG257 had a genome size of 2.78 Mb, consisting of 118 contigs. The genome contained 2351 coding genes, 4 rRNA genes, and 32 tRNA genes (Figure 2B). Gene annotation of MAG257 identified a urease gene cluster (*ureA*, *ureB*, *ureC*, *ureD*, *ureE*, *ureF*, and *ureG*) as well as a urea transporter gene cluster (*urtA*, *urtB*, *urtC*, *urtD*, and *urtE*) (Figure 2C).

Metabolic pathway analysis indicated that MAG257 could hydrolyze and utilize urea and starch to produce amino acids (AAs), propionate, and lactate (Figure 2D). According to the functional genes annotated, we proposed the following urea metabolism pathway for MAG257. Nickel (Ni) is transported into the cell via ferric citrate transport protein D (fecD) and TonB, where it is taken up by the urease accessory protein complex UreDEFG, which transfers and incorporates Ni into the active centre of the urease structural protein complex UreABC, thereby activating urease. Urea, transported by the UrtABCDE system, is hydrolyzed by the active urease to produce ammonia. Ammonia is then used in the synthesis of glutamate and glutamine via glutamate dehydrogenase A (GdhA), glutamate synthase B (GltB), glutamate synthase D GltD, and Glutamine synthetase A (GlnA), providing precursors for AA synthesis. The 2‐ketoglutarate used for ammonia metabolism is from the pyruvate produced from starch.

Phylogenetic analysis using GTDB‐Tk placed MAG257 close to unclassified members of the family Succinivibrionaceae, suggesting that it represents a new uncultured genus (Figure 2E). To confirm the high ureolytic activity of MAG257 urease, the active‐based metaproteomic analysis was performed. Urease subunit UreC peptides were identified in the protein fraction with the highest urease activity, separated by gel filtration chromatography. Liquid chromatography‐tandem mass spectrometry (LC‐MS/MS) analysis of this fraction identified 79 UreC peptides from MAG257, covering 57% of the UreC protein (Figure 2F), indicating that MAG257 exhibits high ureolytic activity. Finally, the relative abundance of MAG257 was assessed across six ruminant species including dairy cattle, goats, sheep, yaks, buffaloes, and deer. It was showed that MAG257 was present in the rumen of different ruminants but no significant change (*p* > 0.05) (Figure 2G).

### Interactions of MAG257 urease subunits

**Figure 3:**
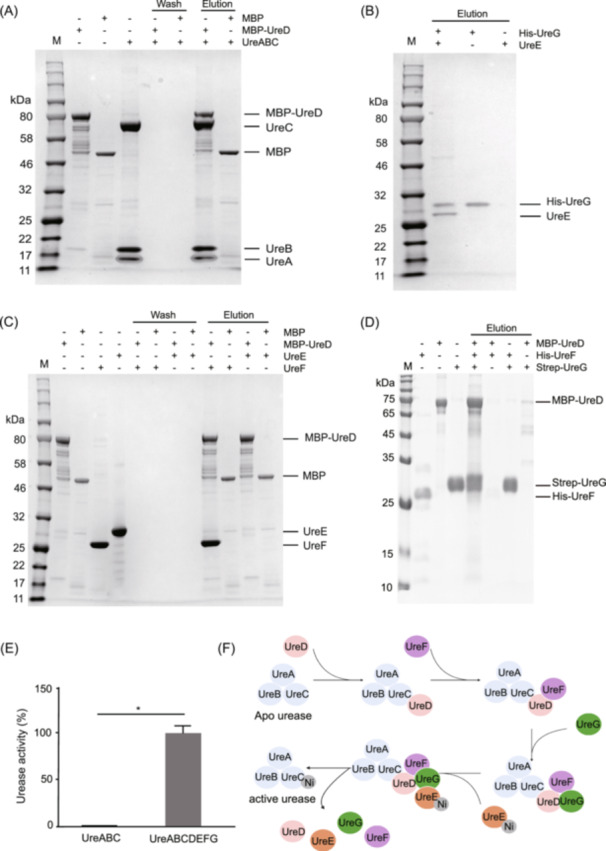
Interaction among MAG257 urease proteins using a pull‐down assay. (A) SDS‐PAGE analysis of MBP‐UreD interactions with UreABC. M, protein marker. (B) SDS‐PAGE analysis of His‐UreG interactions with UreE. (C) SDS‐PAGE analysis of MBP‐UreD interactions with UreE and UreF. (D) SDS‐PAGE analysis of Strep‐UreG interactions with MBP‐UreD and His‐UreF. (E) Urease activity comparison between UreABC and UreABCDEFG complexes. *n* = 3; *p < 0.05 indicates a significant difference. (F) Schematic diagram of the urease activation process. UreD is the first accessory protein to bind to UreABC. UreF then binds to UreD, facilitating UreG binding to the UreDF complex. Finally, UreE delivers nickel ions to UreABC, completing urease activation. MBP, maltose‐binding protein; SDS‐PAGE, sodium dodecyl sulfate‐polyacrylamide gel electrophoresis; UreA, urease A; UreB, urease B; UreC, urease C; UreD, urease D; UreE, urease E; UreF, urease F; UreG, urease G.

The urease subunit proteins UreABC, UreD, UreE, UreF, and UreG were successfully expressed and purified. To investigate the interactions between these subunits, UreABC, UreE, and UreF were incubated with MBP‐tagged UreD in a column, and eluted with maltose. Sodium dodecyl sulfate‐polyacrylamide gel electrophoresis (SDS‐PAGE) analysis revealed that UreD could bind to both UreABC and UreF but not to UreE (Figure 3A,C). His‐tagged UreG was mixed with UreE, UreD, and UreF, and subsequent SDS‐PAGE analysis showed that UreG could bind to UreE as well as the UreD‐UreF complex (Figure 3B,D). Further, the urease activity of the UreABCDEFG complex was significantly higher than that of UreABC alone (*p* < 0.05; Figure 3E), indicating that UreD, UreE, UreF, and UreG are essential for full urease activation. In Figure 3F, we propose an activation model for UreABC facilitated by the interactions of UreDEFG for Ni transfer based on the above findings.

### Cryo‐EM protein structure of the MAG257 ureC

The MAG257 urease UreABC complex, composed of UreA, UreB, and UreC subunits, was initially examined by negative staining (Figures S2 and S3A). The fraction displaying the best particle distribution was immediately used to prepare Cryo‐electron microscopy (cryo‐EM) grids, which were plunge‐frozen in liquid ethane. Representative 2D class averages showed a leaf‐like structure, with a central “T” line separating the particle into three distinct portions (Figure S3A). After 3D classification, the predominant class, containing 742,156 particles, exhibited the best overall density and was selected for further 3D refinement. The resolution improved from 3.95 to 3.1 Å (Figure S3B), enabling the construction of a reliable atomic model (Table S1).

**Figure 4:**
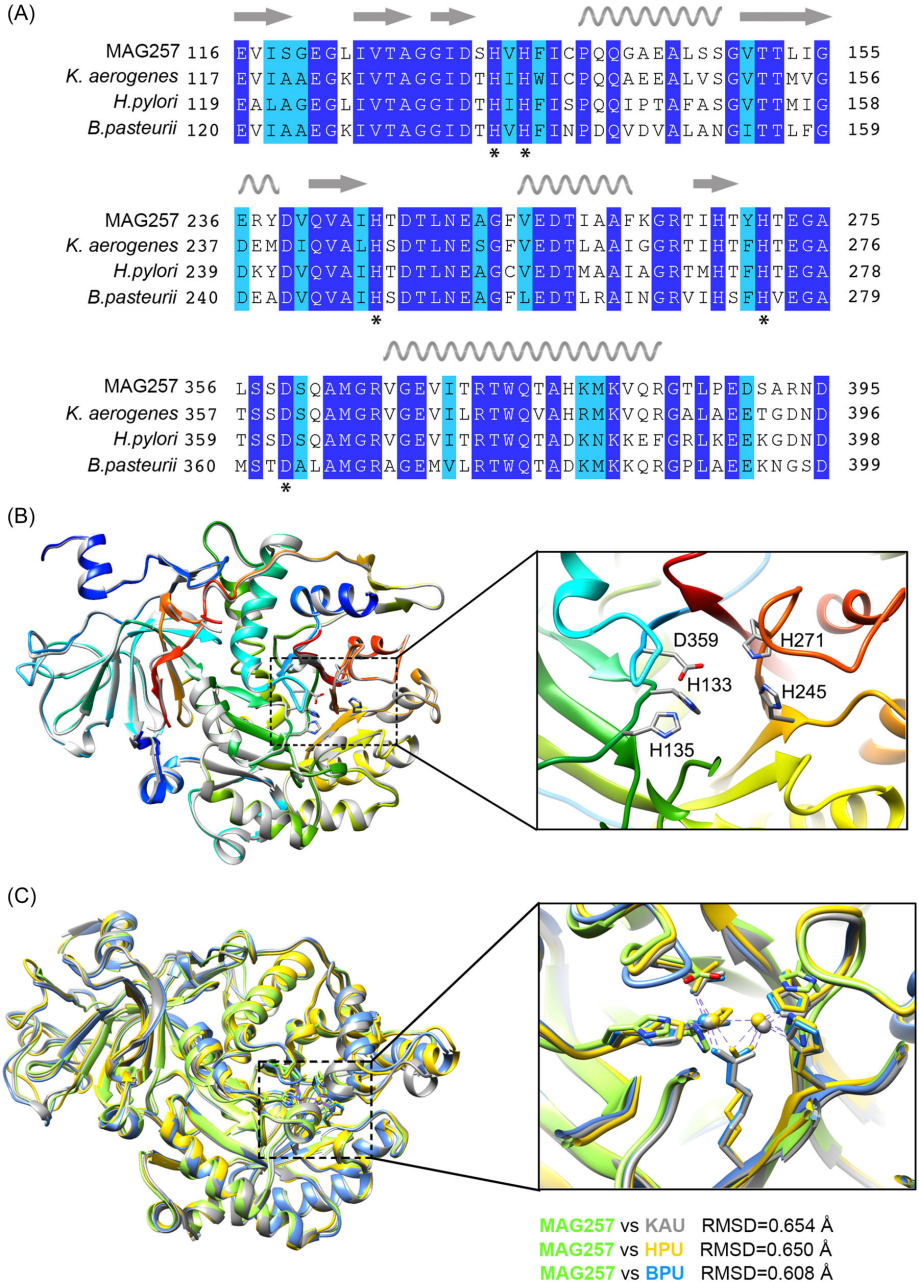
Protein structural characteristics of MAG257 urease. (A) Sequence alignment of UreC from MAG257, *K. aerogenes*, *H. pylori*, and *B. pasteurii*. Identical residues are highlighted in the dark blue, and similar residues are shown in light blue. Secondary structure elements are displayed above the sequence based on the MAG257 UreC structure, in which the wavy line represents alpha helix and the arrow represents beta sheet. Active site residues are marked with an asterisk (*) below the aligned sequences. (B) Atomic structure of MAG257 UreC resolved by cryo‐EM single‐particle analysis. Superimposition of two UreC subunits, one in gray and the other in rainbow colors (blue to red indicating the N‐ to C‐terminus), shows an RMSD of 0.001 Å. The active site residues identified in the sequence alignment are highlighted and shown in ball‐and‐stick representation. (C) Superimposition of atomic models from MAG257 UreC (light green), *K. aerogenes* UreC (gray), *H. pylori* UreC (yellow), and *B. pasteurii* UreC (light blue).

UreC is the most important subunit due to the presence of urease active center. So we focused on the structure of UreC which was essential for the future regulation of activity. A multiple sequence alignment revealed that the catalytic residues were conserved (Figure 4A). The key residue Lys216, responsible for bridging and stabilizing the two Ni atoms, was not carbamylated. Conserved residues His245 and His271 were predicted to coordinate Ni (1), while His133, His135, and Asp359 were predicted to coordinate Ni (2) (Figure 4B). The densities for the two Ni²⁺ ions were not observed in the cryo‐EM map, likely due to the lack of posttranslational modifications (e.g., Lys216 carbamylation) and the moderate resolution. Superimposing the structures of all four ureases yielded a root mean square deviation (RMSD) between 0.608 and 0.654 Å (Figure 4C), indicating relatively conserved structural folding.

### Screening of urease inhibitors by molecular docking

**Figure 5:**
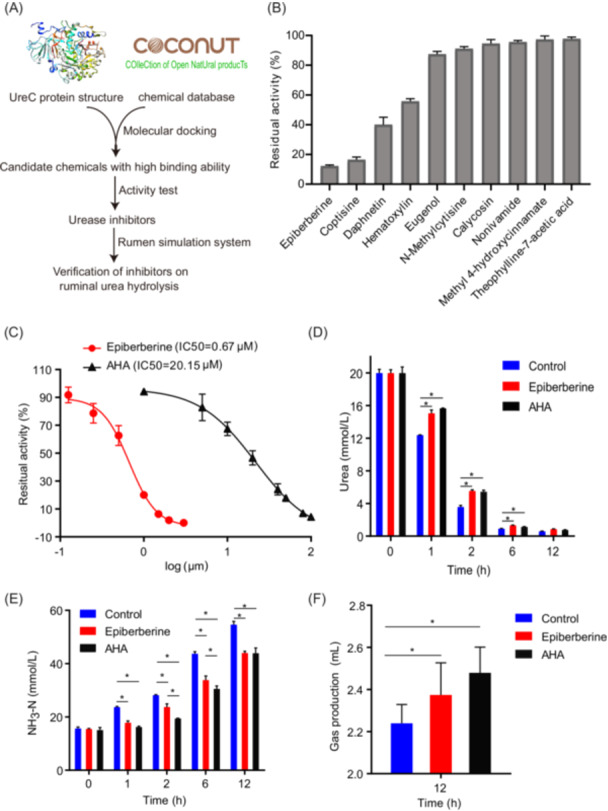
Screening and effects of epiberberine on urease activity and urea metabolism in a rumen simulation system. (A) Diagram illustrating the screening process of urease inhibitors from natural compounds. (B) Effects of selected natural compounds on the activity of ruminal microbial urease. (C) Dose‐dependent inhibition of ruminal microbial urease with different concentrations of epiberberine. (D) Impact of epiberberine on the urea disappearance rate in a rumen simulation system. (E) Effect of epiberberine on NH₃‐N production in a rumen simulation system. (F) Influence of epiberberine on total gas production in a rumen simulation system. AHA is used as a reference inhibitor throughout the assays. *p < 0.05 denotes a significant difference compared to the control group. AHA, acetohydroxamic acid.

Molecular docking against the active center of the MAG257 UreC protein was used for urease inhibitor screening based on their binding energies (Figure 5A and Figure S4). The residual activities of ruminal microbial urease after inhibition by nine natural compounds are shown in Figure 5B. Among these, epiberberine, daphnetin, and hematoxylin reduced activity by 40% or more, with epiberberine demonstrating the strongest inhibitory effect (~90%). Further investigation into the half maximal inhibitory concentration (IC50) values of epiberberine and acetohydroxamic acid (AHA) (positive control) against ruminal microbial urease revealed that epiberberine exhibited significantly higher inhibition potency. The IC50 value for epiberberine was 0.67 μM, which is 30 times lower than that of AHA (Figure 5C).

### Integral urea hydrolysis and fermentation of microbial community affected by epiberberine

To explore the potential application of epiberberine in ruminants, its effect on urea metabolism in the ruminal microbial community was assessed using a rumen simulation system. The residual urea concentration in the epiberberine group was 17.73% higher than in the control group after 1 h of incubation (*p* < 0.05), indicating that epiberberine slowed down the decomposition of urea (Figure 5D). NH₃‐N levels in both the epiberberine and AHA groups were significantly lower than those in the control group (*p* < 0.05; Figure 5E). Additionally, the total microbial fermentation gas produced by the epiberberine groups was significantly higher than control group at the end of fermentation (*p* < 0.05; Figure 5F).

### Binding interaction of epiberberine with urease

**Figure 6:**
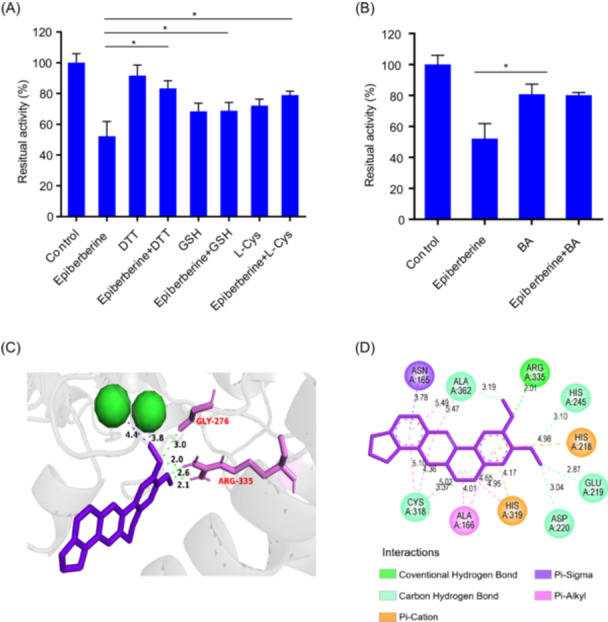
Characterization of epiberberine binding with MAG257 UreC. (A) The protective effects of thiol‐containing compounds (DTT, GSH, and l‐Cys) on the inhibition of ruminal microbial urease by epiberberine. (B) Effect of boric acid on the inhibition of epiberberine on ruminal microbial urease. (C) Interaction between epiberberine and the active site of UreC. Epiberberine is shown in purple, two nickel ions as green spheres, key residues (Gly276 and Arg335) in pink, and hydrogen bonds as green dotted lines. Numbers indicate bond distances in Ångstroms. (D) Visualization of the interactions between epiberberine and UreC. *p < 0.05 indicates a significant difference compared to control. BA, boric acid; DTT, dithiothreitol; GSH, glutathione; l‐Cys, l‐cysteine.

To investigate the binding site of epiberberine with urease, three thiol‐containing compounds including DTT, l‐Cys, and GSH were used to assess whether epiberberine interacts with the cysteine sulfhydryl group in the urease active center. The ruminal microbial urease activity was inhibited by 52.2% by epiberberine in this assay. However, when epiberberine was combined with DTT, l‐Cys, or GSH, urease activity was higher (*p* < 0.05), indicating that epiberberine likely interacts with the mobile flap motif in the active center (Figure 6A). Boric acid was used to assess whether epiberberine inhibits urease by interacting with Ni. Boric acid completely blocked the inhibitory activity of epiberberine (*p* < 0.05; Figure 6B). This suggests that epiberberine interacts with Ni at the urease active site.

Molecular docking was used to characterize the binding profile of epiberberin and the active center of UreC. Epiberberine's two methoxy groups helped orient the molecule within the urease active cavity. The oxygen atom of the 2‐O‐CH₃ group formed hydrogen bonds with the oxygen atom of Gly276 and the NH group of Arg335 (Figure 6C). Additional hydrogen bonds were formed between the 3‐O‐CH₃ group and two NH groups of Arg335. Five binding models were observed, with the closest distances between epiberberine (CH group of 2‐O‐CH₃) and the two Ni ions in UreC being 4.4 Å and 3.8 Å, respectively. Key amino acid interactions are illustrated in Figure 6D. Ala362 interacted with epiberberine via Pi‐alkyl interactions with the C and D rings, and a carbon‐hydrogen bond with the 2‐O‐CH₃ group. His245 and His318 formed carbon‐hydrogen bonds with the 3‐O‐CH₃ group, while Glu219 and Asp220 interacted with the CH group of 3‐O‐CH₃. Notably, Cys318, a key residue regulating the mobile flap of urease, interacted with the B, C, and D rings via Pi‐alkyl interactions and formed a carbon‐hydrogen bond with the B ring. A Pi‐sigma interaction was observed between Asn165 and the D ring.

## DISCUSSION

In this study, we developed a comprehensive methodology to identify novel microbial regulators by combining core protein identification, protein structure analysis, molecular docking, and functional verification. This approach successfully identified epiberberine, a natural urease inhibitor, and demonstrated its inhibitory effect on urease activity through in vitro fermentation assays. Our results indicate that this method holds significant potential as a practical and valuable approach for modulating targeted microbiome function in areas such as animal gut health (e.g., diarrhea‐related pathogens, methane‐producing archaea, and acid‐producing bacteria causing rumen acidosis), human gut health (e.g., *Enterobacter* and *Clostridia* related chronic inflammatory), and environmentally sustainable development (e.g., microbes driven greenhouse effect).

Our methodology mirrors previous successful approaches for screening microbial inhibitors. For instance, 3‐nitrooxypropanol (3‐NOP) was identified through molecular docking as a potent inhibitor of methane production by targeting methylcoenzyme M reductase in *Methanothermobacter marburgensis* [18]. Subsequent in vivo trials showed that 3‐NOP reduced methane emissions by 30% without affecting feed intake or milk production in dairy cows [19]. At present, 3‐NOP, marketed as Bovaer, has been successfully approved for use as a methane‐reducing feed additive in several countries. This highlights the utility of molecular docking‐based approaches for identifying effective inhibitors that can be applied in the field, including urease inhibitors such as epiberberine.

The study of core microbiomes has gained significant attention in recent years, particularly for understanding key microbial functions within specific environments [20]. In our study, we extended this definition to include microbial activity, specifically urease activity, in the identification of core microbiomes. Previous research has highlighted the variability in rumen core microbiomes due to factors such as host species, diet, and geography [21]. In this study, we increased the diversity and robustness of our sample set. This broad sample diversity enabled us to accurately identify nine core urease genes in the rumen with higher abundance and prevalence. A core urease‐located genome, MAG257, was identified through sequence alignment with core urease genes. The active‐based metaproteomics analysis also revealed the high urease activity of MAG257. In this study, we defined the core enzyme as one with high abundance, prevalence, and activity in the microbiome.

The functional gene annotation of MAG257 provides new insight into its functional role. The metabolic pathways of MAG257 indicated its ability to utilize urea and starch to produce AAs, propanoate, and lactate. The rapid generation of energy and carbon skeletons from starch enhances the efficiency of urea nitrogen utilization during the ammonia assimilation process. We used cryo‐EM to resolve the structure of the MAG257 UreC at 3.1 Å resolution. The number of subunits in urease structural proteins can vary among bacterial species, but the overall composition of these subunits remains related across different organisms [22]. The ureases of *Klebsiella aerogenes*, *Bacillus pasteurii*, *and Helicobacter pylori* are among the most thoroughly studied bacterial ureases. Specifically, the ureases of *K. aerogenes* and *B. pasteurii* are structured as (αβγ)_3_ complexes, whereas *H. pylori* urease forms a [(αβ)_3_]_4_ complex [22, 23]. Multiple sequence alignment of the catalytic subunit from MAG257 with those from these three bacteria further confirms the homology of the α subunits in urease.

Natural plant‐derived inhibitors, including essential oils, tannins, and saponins, have shown promise in modulating rumen microbial activity and nitrogen metabolism [24]. Our study identified epiberberine as a rumen microbiota urease inhibitor, with an IC50 value of 0.67 μM, which is significantly lower than other natural inhibitors like biochanin A (320 μM) [25] and coptisine (2.45 μM) [26]. This suggests that epiberberine is a strong inhibitor for rumen microbiota urease, capable of modulating urea hydrolysis even at low concentrations.

The mechanism of urease inhibition typically involves one of three patterns: (1) direct interaction with Ni in the active site, as seen with thiourea and its analogs; (2) interaction with the mobile flap covering the active site, as with 1,4‐benzoquinone and Au ions [27]; or (3) interaction with both the active site and mobile flap, as observed with coptisine [28]. Epiberberine follows the third mechanism, interacting with key residues such as Gly276, His245, His218, His319, and Cys318, which are located in the active site and flap region of the urease enzyme. Our results from thiol‐containing compound assays further support this, showing that epiberberine inhibits urease by binding to the sulfhydryl group of cysteine in the flap region, preventing proper access to the active site. The well‐known urease inhibitor acetohydroxamic acid can only bind to the active center to form a CH_3_–CO–NHOH complex [29], which explains the observed lower inhibition activity compared to epiberberine.

Overall, while our study highlights the promising potential of epiberberine as a urease inhibitor, future research is needed to fully understand its effects by cattle feeding trial. Additionally, more efficient inhibitors can be screened using the constructed urease protein and other more varied and abundant chemical databases.

## CONCLUSION

In this study, we developed and validated a novel methodology for regulating microbial community functions. This approach integrated core functional protein identification, protein structure analysis, molecular docking, and functional verification using a simulation model. Through this method, we successfully identified the core ruminal urease in dairy cows and discovered that the natural compound epiberberine exhibited a strong inhibition of rumen microbiota urease activity. These results suggest that this methodology is feasible and provides a powerful tool for regulating microbiota functions.

## METHODS

### Rumen fluid sample collection and microbial DNA extraction

Rumen fluid samples were collected from 88 Holstein dairy cows fed 10 different types of diets from various farms across China using oral stomach‐tubing. Detailed sample information is provided in Table S2. Total microbial DNA was extracted from rumen samples using the Cetyltrimethylammonium Bromide (CTAB) and bead‐beating method as described previously [30]. DNA integrity was confirmed via 1% agarose gel electrophoresis and quantified using a NanoDrop One spectrophotometer (Thermo Fisher Scientific).

### Urease gene sequencing and core analysis

The urease gene (*ureC*) was amplified, sequenced, and analyzed as described by our previous study [31]. Briefly, the primer set is UreC‐F (5′‐TGGGCCTTAAAATHCAYGARGAYTGGG‐3′) and UreC‐R (5′‐GGTGGTGGCACACCATNANCATRTC‐3′). Sequencing was performed on the Illumina MiSeq platform (2 × 300 bp). OTUs were generated at a 97% similarity threshold using USEARCH (v6.1) [32]. Taxonomic classification of *ureC* genes was assigned using GraftM (v0.14.0) [33] with a likelihood cutoff value of 0.75 based on an *ureC* gene database according to the previous study [31]. Alpha and beta diversity analyses were conducted in QIIME (v1.9) [34]. Core *ureC* OTUs were defined as having a relative abundance greater than 1% and sample coverage exceeding 85%. Phylogenetic analysis of core *ureC* OTUs was performed in MEGA X (v10.2.6) [35].

### Identification of core MAG with urease genes

Ten DNA samples (one from each diet) were randomly selected for shotgun metagenomic library construction using the TruSeq DNA PCR Free Kit (Illumina), and sequencing was carried out on the Illumina HiSeq. 2500 platform (2 × 150 bp). Low‐quality reads were filtered using Trimmomatic (v0.32) [36], and *de novo* assembly was conducted using Megahit (v1.2.9) [37]. MAGs were generated with MetaBat2 in MetaWrap (v1.2) [38]. Core *ureC* genes were aligned with the MAGs using blastn, and core MAGs containing *ureC* with 100% sequence homology were identified. The selected core MAG (MAG257) was annotated using Prokka (v1.13) [39], and a circular genome diagram was generated using Circos (v0.69‐9) [40]. Functional annotation of MAG257 was carried out with eggNOG‐mapper (v2.1.9) [41], and genome quality was assessed with CheckM (v1.0.18) [42]. Taxonomic identification of MAG257 was completed using GTDB‐Tk (v2.4.0) based on genome taxonomy database (GTDB) (v109) [43]. Phylogenetic analysis of Succinivibrionaceae family members was performed in MEGA X (v10.2.6) [35].

To verify the activity of MAG257 urease, activity‐ and enrichment‐based metaproteomics were employed by our previous study [44]. Briefly, rumen microbial crude protein was extracted via cryomilling, and gel filtration chromatography was used to separate proteins. Urease activity in each fraction was measured, and the fraction with the highest activity was subjected to SDS‐PAGE and LC‐MS/MS. The peptides were identified using PEAKS Studio (v12.5) software, comparing a rumen metagenome‐derived protein database with MAG257 UreC protein.

To assess the prevalence of MAG257 across different ruminants, the published rumen metagenomes from dairy cattle, goats, sheep, yaks, buffalo, and deer [45] were downloaded and quality filtered. The relative abundance of MAG257 in different ruminants was calculated using the Salmon in MetaWrap (v1.2) quant_bins module [38], with the assembled genomes as input. Relative abundance of MAG257 was expressed as counts per million reads (CPM).

### Expression and purification of MAG257 urease proteins

The urease genes *ureABCDEFG*, *ureA*, *ureB*, *ureC*, *ureD*, *ureE*, *ureF*, *and ureG* were amplified by PCR using rumen total microbial DNA as a template. Primers are listed in Table S3. PCR products were purified using the Agarose Gel DNA Extraction Kit (Tiangen, Beijing, China). Genes were digested with corresponding restriction enzymes and ligated into vectors (NEB) (Table S4). Maltose‐binding protein (MBP) tags, ureD, and linearized pETite were assembled via Gibson Assembly Master Mix (NEB, Ipswich, MA), with MBP sequences obtained from the pmal‐C5X plasmid by PCR (Table S3). Recombinant plasmids were transformed into *E. coli* XL10 (Weidi Biotechnology) and extracted using the TIANprep Mini Plasmid Kit (Tiangen). Plasmid sequences were confirmed by Sanger sequencing (ABI 3500, Thermo Fisher Scientific).

pET‐UreC&A and pET28‐UreB were co‐transformed into *E. coli* BL21, while other plasmids were separately transformed into *E. coli* BL21. Protein expression was induced with 0.5 mM isopropyl β‐D‐1‐thiogalactopyranoside (IPTG) overnight. UreABC protein complex was purified using Ni‐NTA resin (Thermo Fisher Scientific) and further purified by Source Q anion exchange chromatography on an AKTA Protein Purification System (GE). The anion exchange column was pre‐equilibrated with solution A (20 mM HEPES, pH 7.5), and the target protein was eluted by solution B (20 mM HEPES, pH 7.5, 1 M NaCl) with the gradient of NaCl from 0 to 1 M. Final purification was performed using gel filtration chromatography on AKTA Pure equipped with Superose 6 Increase 10/300GL (GE) with elution buffer (20 mM HEPES, pH 7.5, 150 mM NaCl) and a flow rate of 0.5 mL/min.

The MBP‐UreD protein was purified using the PurKine™ MBP‐Tag Dextrin Resin 6FF (Abbkine) following the manufacturer's protocol. The UreABCDEFG, UreF, UreE, and UreG proteins were purified using the HisTrap FF column (GE Healthcare).

### Interaction of urease proteins analyzed by pull‐down assay

The purified MBP‐UreD protein was immobilized on PurKine™ MBP‐Tag Dextrin Resin 6FF (Abbkine) to serve as the bait protein. Purified UreABC complex, UreF, and UreE proteins were then added individually to the resin‐bound MBP‐UreD. After incubation at 4°C for 2 h, the resin was washed with a washing buffer (20 mM Na₃PO₄, 500 mM NaCl, pH 7.4). The bound proteins were eluted using an elution buffer (20 mM Na₃PO₄, 500 mM NaCl, 10 mM maltose, pH 7.4) and analyzed by SDS‐PAGE.

For the interaction between His‐tagged UreG and UreE, His‐UreG was used as the bait protein. The Ni‐NTA resin (Thermo Fisher Scientific) was washed with a washing buffer containing 20 mM Na₃PO₄, 500 mM NaCl, and 10 mM imidazole (pH 7.4). The bound proteins were eluted with an elution buffer (20 mM Na₃PO₄, 500 mM NaCl, 200 mM imidazole, pH 7.4) and analyzed by SDS‐PAGE. Urease activity of the UreABC and UreABCDEFG complexes was measured based on ammonia production, as described by our previous study [44].

### Cryo‐EM analysis of MAG257 UreC protein structure

Freshly purified UreABC protein was diluted to approximately 1 mg/mL in a buffer containing 20 mM HEPES (pH 7.39) and 150 mM NaCl. A 4 μL aliquot of the diluted sample was applied to a glow‐discharged Quantifoil grid (Au 1.2/1.3 400 mesh). The grids were blotted for 3 s at 100% humidity and rapidly plunged into liquid ethane using a Vitrobot Mark IV (Thermo Fisher Scientific).

Cryo‐EM data collection was performed on a Titan Krios G3i electron microscope (Thermo Fisher Scientific), operated at 300 kV, equipped with a K3 direct electron detector, and a BioQuantum energy filter (Gatan) set to a slit width of 20 eV. All movies were recorded in super‐resolution mode at a nominal magnification of 130,000×, yielding a pixel size of 0.54 Å. The total electron dose was set to 50 e‐/Å², with a defocus range between −1.0 and −2.0 μm.

A total of 2822 dose‐fractionated movies were subjected to motion correction using MotionCor2 (v1.4.4) [46] with dose weighting, resulting in a final pixel size of 1.08 Å. The contrast transfer function (CTF) parameters, including defocus values and astigmatism, were estimated using CTFFIND (v4.1.14) [47]. A total of 2726 micrographs were selected for further analysis. Auto‐picked particles were subjected to several rounds of 2D classification in Relion (v3.1) [48]. An initial 3D model was generated ab initio in Relion (v3.1), and after 2D classification, 1,783,732 particles were retained. From these, 742,156 particles, displaying well‐defined secondary structural features and optimal density distribution, were selected for 3D auto‐refinement. A final resolution of 3.1 Å was determined using the Fourier shell correlation at the 0.143 criterion.

Model building of the urease began with the rigid‐body fitting of individual protein structures, predicted by AlphaFold (v2) [49], into the cryo‐EM density map using UCSF ChimeraX (v1.9) [50]. Manual model refinement was performed using Coot (v1.1) [51]. Further refinement was carried out using the real‐space refinement module in Phenix (v1.20) [52], with restraints applied to secondary structure, Ramachandran plots, rotamer outliers, and C‐beta deviation to optimize model geometry. The model was iteratively refined in Coot (v1.1) and Phenix (v1.20). Model validation was performed using MolProbity (v4.5.1) [53] (Table S1), and figures were generated using ChimeraX (v1.9) and PyMOL (v2.5.0).

### Screening of urease inhibitors by molecular docking

The protein structure model of MAG257 UreC and the COCONUT natural compounds database [54] were prepared using AutoDock (v4.2) and AutoDock Tools (v1.5.6). The cubic grid box for the blind docking area of UreC active center was set to a size of 108 × 126 × 122 Å. Docking was performed using the Lamarckian genetic algorithm with a population size of 150, a maximum of 2,500,000 energy evaluations, and a maximum of 27,000 generations.

The ruminal microbial crude urease solution was prepared as described by our previous study [26]. The top 10 compounds from the molecular docking were obtained from Topscience (Shanghai), dissolved in dimethyl sulfoxide (DMSO), and further diluted with 50 mM HEPES buffer (pH 7.5). Urease activity was determined as described by our previous study [26]. AHA was used as a positive control. To further evaluate epiberberine's inhibition of urease, its IC50 was measured, with AHA serving as a standard inhibitor. IC50 values were calculated using nonlinear regression analysis in GraphPad Prism (v6).

### Changes of microbial fermentation influenced by epiberberine

A Rumen Simulation System was employed to assess the inhibitory effect of epiberberine on urea hydrolysis and its influence on fermentation processes. Anaerobic media and diluents were prepared following established protocols [26]. Each Hungate tube received 0.5 mL of 1.2 mM epiberberine or acetohydroxamic acid (AHA) and 0.5 mL of 1.2 M urea solution, followed by incubation at 39°C in an anaerobic chamber (Plas‐Labs). Samples (200 μL) were taken at 0, 1, 2, 6, and 12 h using a needle and syringe. Ammonia‐N concentration was measured using an Ammonia Assay Kit (Jiancheng), while urea levels were determined using a Urea Detection Kit (Jiancheng). Gas pressure in the tubes was recorded at 12 h with a digital pressure gauge (ConST), and used to calculate gas production according to the previous study [25].

### Binding site of epiberberine

To investigate the potential binding site of epiberberine on ruminal microbial urease, its activity was evaluated in the presence of thiol‐containing compounds, including dithiothreitol (DTT), l‐cysteine (l‐Cys), and glutathione (GSH). Initially, 40 μL of urease solution was combined with 40 μL of epiberberine solution (diluted in 50 mM HEPES buffer, pH 7.5) in 96‐well plates and incubated at room temperature for 10 min. Subsequently, DTT, l‐Cys, or GSH (diluted in 50 mM HEPES buffer, pH 7.5) were added to the wells and incubated at 37°C for 30 min. The final concentrations of epiberberine and thiol compounds were 1 μM and 0.25 mM, respectively. The effect of boric acid on microbial urease activity was also evaluated. A boric acid solution (dissolved in 50 mM HEPES buffer, pH 7.5) was added to the pre‐incubated mixture of epiberberine and urease for 30 min at 37°C. The final concentrations of epiberberine and boric acid were 1 μM and 0.25 mM, respectively. The HEPES buffer was used for the control during thiol‐containing compounds and boric acid tails. Binding interactions between epiberberine and amino acids in the MAG257 UreC active site were visualized using PyMOL (v2.5.0) and Discovery Studio (v4.5).

### Statistical analysis

Differences in gene expression or microbial relative abundance were analyzed using the Kruskal–Wallis test. Repeated measures ANOVA followed by Tukey's post hoc test was employed to compare rumen fermentation data in SAS Studio (v3.81). Statistical significance was set at *p* < 0.05.

## AUTHOR CONTRIBUTIONS

**Shengguo Zhao**: Conceptualization; methodology; software; data curation; investigation; validation; formal analysis; project administration; resources; visualization; funding acquisition; writing—original draft; writing—review and editing; supervision. **Huiyue Zhong**: Methodology; data curation; validation; formal analysis; visualization; writing—original draft; writing—review and editing. **Yue He**: Methodology; data curation; formal analysis. **Xiaojiao Li**: Methodology; validation; visualization; data curation; writing—original draft; writing—review and editing. **Li Zhu**: Methodology; validation; software; formal analysis; data curation; writing—original draft; writing—review and editing. **Zhanbo Xiong**: Methodology; data curation; writing—original draft. **Xiaoyin Zhang**: Methodology; data curation; software; formal analysis. **Nan Zheng**: Resources; writing—review and editing. **Diego P. Morgavi**: Writing—review and editing; supervision; resources; writing—original draft; investigation. **Jiaqi Wang**: Conceptualization; supervision; resources; project administration; funding acquisition; writing—original draft; writing—review and editing.

## CONFLICT OF INTEREST STATEMENT

The authors declare no conflicts of interest.

## ETHICS STATEMENT

The animal procedures were approved by the Animal Care Committee of the Institute of Animal Sciences of the Chinese Academy of Agricultural Sciences (approval number: IAS2020‐82).

## Supporting information


**Figure S1.** Alpha diversity across different diets based on *ureC* gene OTUs.
**Figure S2.** Verification of UreA, UreB, and UreC of MAG257.
**Figure S3.** Reconstruction of the MAG257 UreABC complex.
**Figure S4.** Screening of urease inhibitors by molecular docking.

**Table S1.** Cryo‐EM data collection, refinement, and validation statistics.
**Table S2.** Different diets of dairy cows.
**Table S3.** The primers for different urease genes.
**Table S4.** The plasmids, restriction sites antibiotic resistance, and tags for different urease genes.

## Data Availability

The ureC amplicon sequences and MAG257 genome data are in the National Microbiology Data Center with the number of NMDC10019360 and NMDC60198028, respectively (http://resolve.pid21.cn/13913.11.micro.data.project.NMDC10019360, http://resolve.pid21.cn/13913.11.micro.data.genome.NMDC60198028). The rumen metagenome data from different species of ruminants are from GenBank with the number of PRJNA657455 (https://www.ncbi.nlm.nih.gov/bioproject/PRJNA657455). The data and scripts used are saved in GitHub (https://github.com/zhaoshengguo/imeta.git). Supplementary materials (figures, tables, graphical abstract, slides, videos, Chinese translated version, and update materials) may be found in the online DOI or iMeta Science http://www.imeta.science/.
