# Supplementary material for: Leveraging core enzyme structures for microbiota targeted functional regulation: Urease as an example
Source: Imeta. 2025 Apr 16;4(3):e70032. doi: 10.1002/imt2.70032 (PMC12130578; doi:10.1002/imt2.70032)
Supplement: Supplementary file 1 — Figure S1. Alpha diversity across different diets based on ureC gene OTUs. Figure S2. Verification of UreA, UreB, and UreC of MAG257. Figure S3. Reconstruction of the MAG257 UreABC complex. Figure S4. Screening of urease inhibitors by molecular docking. [file IMT2-4-e70032-s002.docx]

Supporting Information to

**Leveraging core enzyme structures for microbiota targeted functional regulation: Urease as an example**

**Running title:** Leveraging core enzyme structures for microbiota functional regulation

Shengguo Zhao^1#^, Huiyue Zhong^1#^, Yue He^1#^, Xiaojiao Li^1^, Li Zhu^2^, Zhanbo Xiong^1^, Xiaoyin Zhang^1^, Nan Zheng^1^, Diego P Morgavi^3^, Jiaqi Wang^1*^

^1^Key State Laboratory of Animal Nutrition and Feeding, Institute of Animal Sciences, Chinese Academy of Agricultural Sciences, Beijing 100193, China.

^2^Electron Microscopy Centre, Lanzhou University, Lanzhou 730000, China

^3^Université Clermont Auvergne, INRAE, VetAgro Sup, UMR Herbivores, Saint-Genès-Champanelle F-63122, France

^#^These authors contributed equally: Shengguo Zhao, Huiyue Zhong, Yue He

^*^Correspondence: [jiaqiwang@vip.163.com](mailto:jiaqiwang@vip.163.com) (Jiaqi Wang)

**
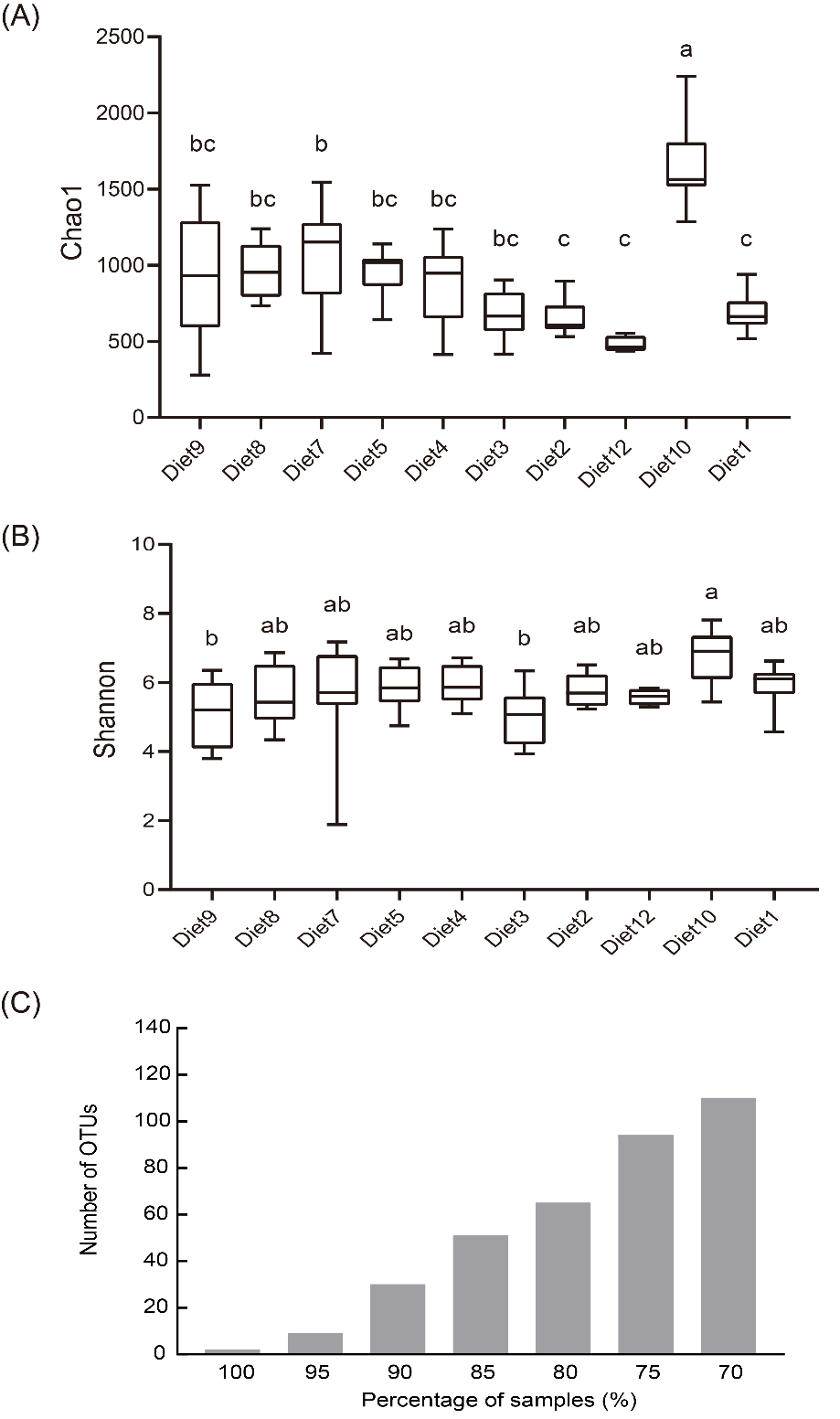
**

**Figure S1 Alpha diversity across different diets based on *ureC* gene OTUs.** (A) The number of *ureC* gene OTUs covering different percentages of samples. (B) Chao1 index of ten diets, with significant differences indicated by different letters. (C) Shannon index of ten diets, with significant differences indicated by different letters.


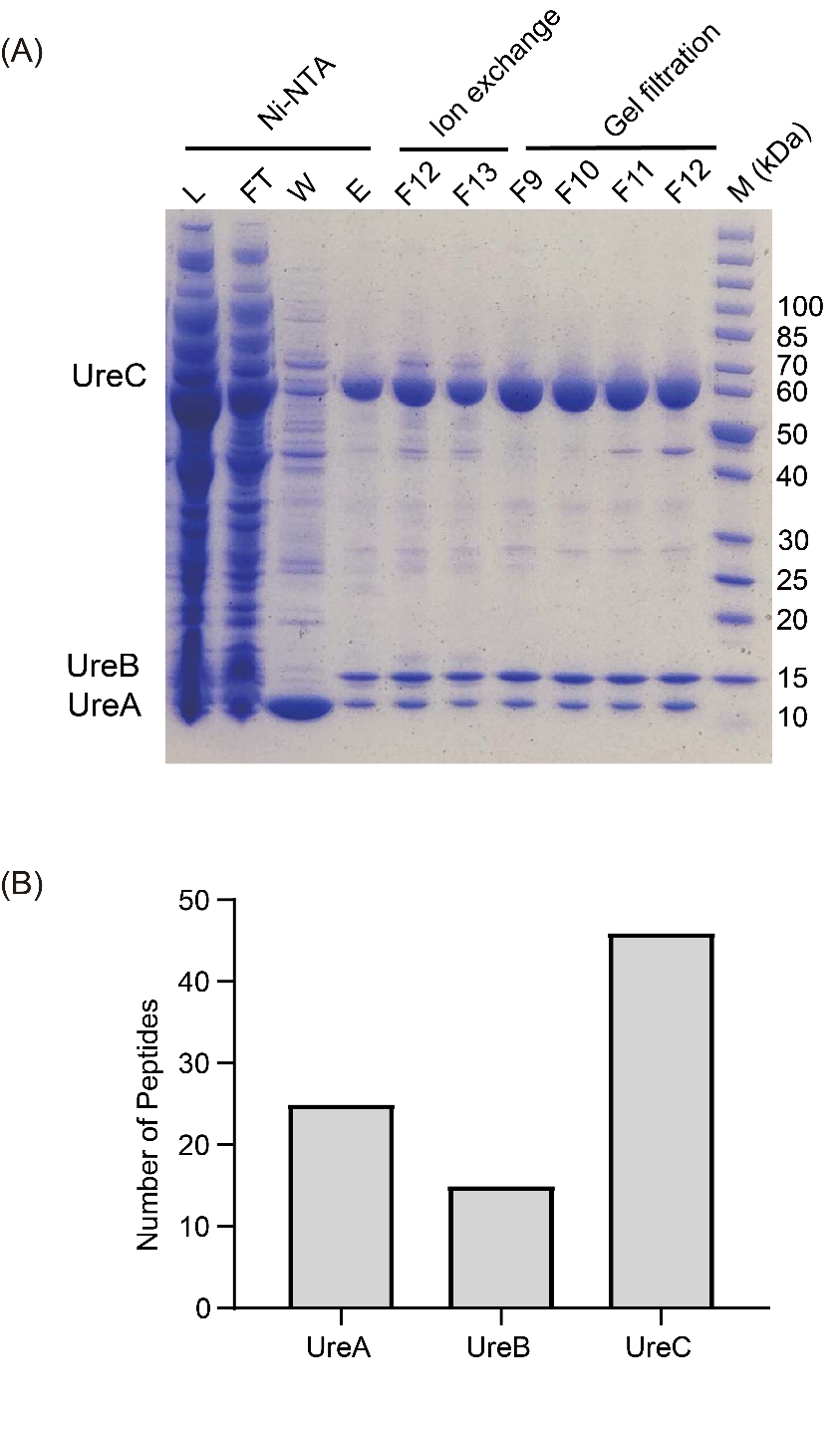


**Figure S2 Verification of UreA, UreB, and UreC of MAG257.** (A) SDS-PAGE analysis of UreA, UreB, and UreC following purification by Ni-NTA, ion exchange, and gel filtration. (B) Number of peptides identified in UreA, UreB, and UreC.

**
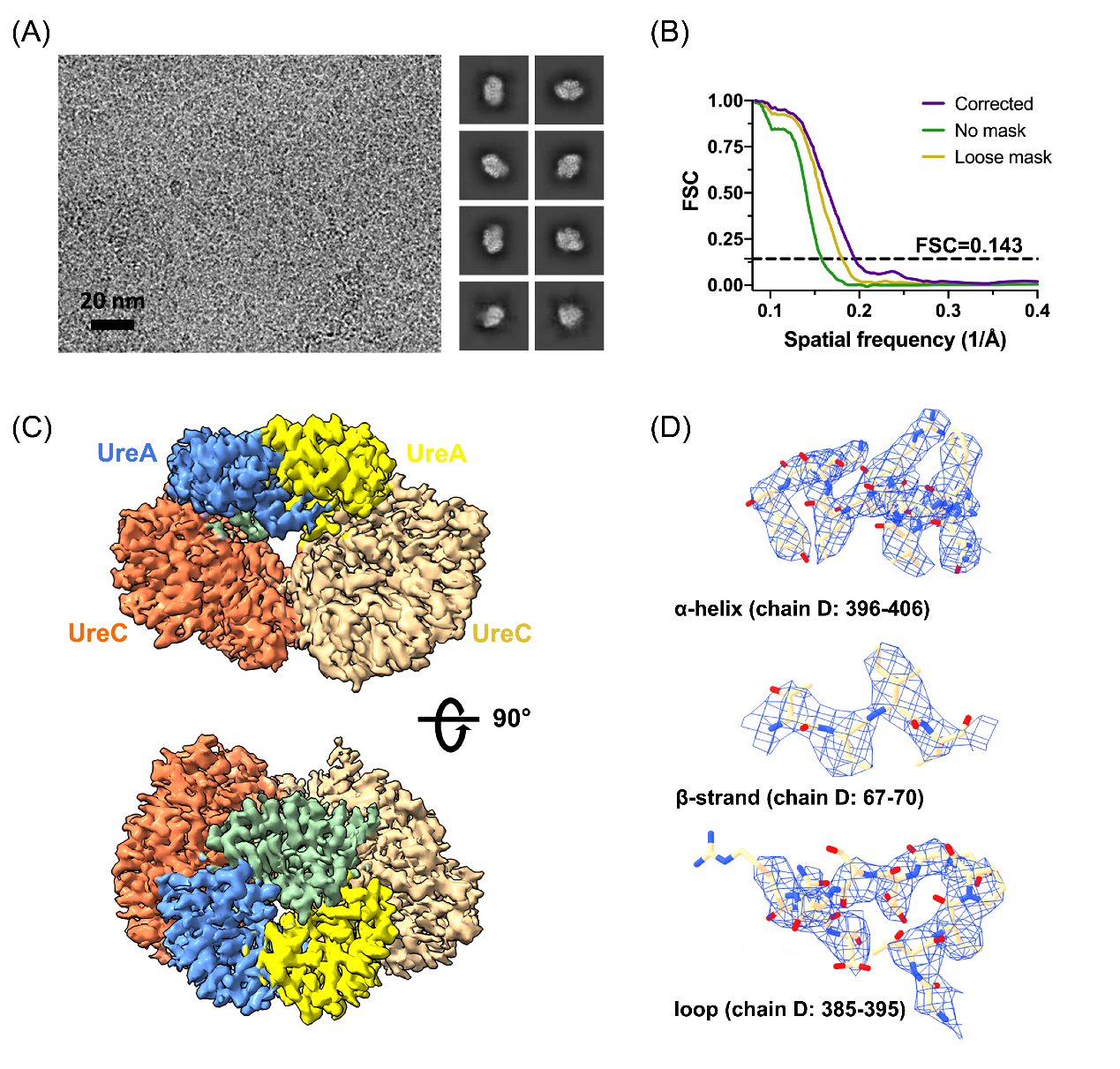
**

**Figure S3 Reconstruction of the MAG257 UreABC complex.** (A) Representative cryo-EM micrograph and 2D class averages of the MAG257 UreABC complex. (B) Gold-standard Fourier shell correlation (FSC) curves with and without masking, indicating a final resolution of 3.1 Å using the FSC 0.143 threshold. (C) 3D reconstruction of the MAG257 UreABC complex shown in surface mode from two different views. Three UreA subunits (blue, yellow, and green) form the upper layer, while two UreC subunits (gold and orange) form the lower layer. No symmetry was imposed during reconstruction. (D) Representative densities of secondary structures in the UreC subunit (chain D).

**
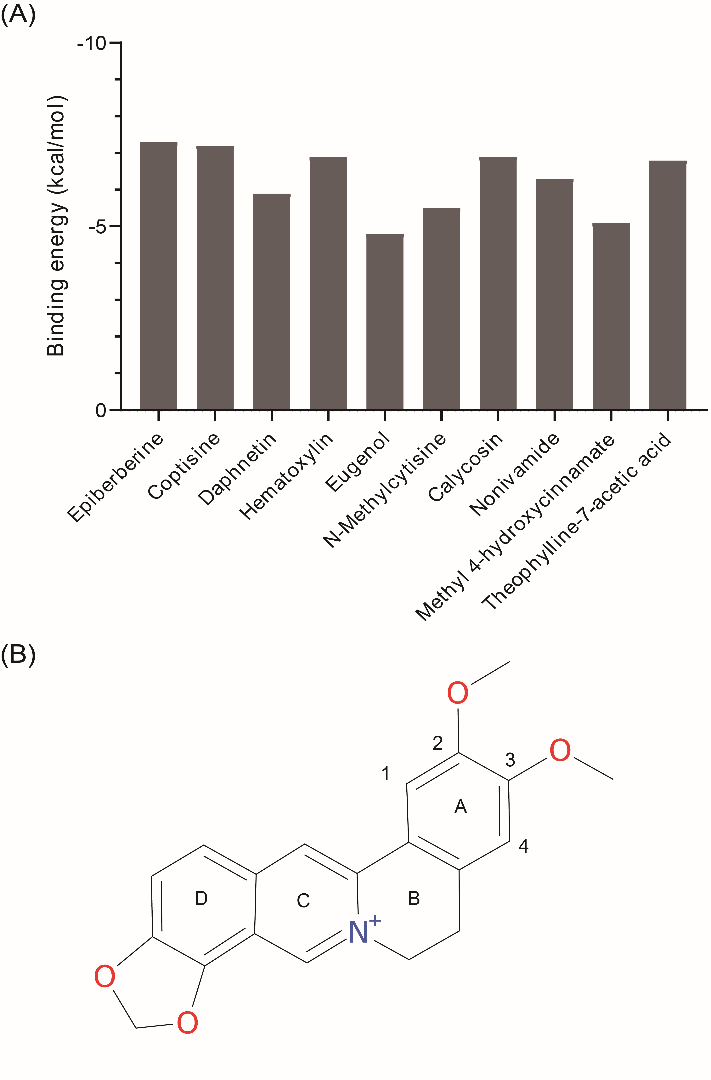
**

**Figure S4 Screening of urease inhibitors by molecular docking.** (A) Binding energy of the ten lowest-energy natural plant compounds screened as potential urease inhibitors. (B) Structural formula of epiberberine, consisting of four aromatic rings (A, B, C, and D) that form the isoquinoline parent structure.
